# Pharmacovigilance study on old drugs repurposed for rare diseases across different indications: the case of fenfluramine

**DOI:** 10.3389/fphar.2025.1682788

**Published:** 2025-09-24

**Authors:** Jiahong Zhong, Zhuomiao Lin, Junling Xue, Xihui Yu

**Affiliations:** ^1^ Department of Clinical Pharmacy, Meizhou People’s Hospital (Huangtang Hospital), Meizhou, China; ^2^ The Second Affiliated Hospital of Shantou University Medical College, Shantou, Guangdong, China

**Keywords:** Lennox-Gastaut syndrome, Dravet syndrome, fenfluramine, Fintepla, drug repurposing, raredisease, FAERS

## Abstract

**Objective:**

As an old drug with a new application in rare diseases with epileptic symptoms, fenfluramine may have potential unrecognized adverse events. Because limited real-world data exist on Lennox-Gastaut syndrome (LGS) and Dravet syndrome (DS) populations, some rare adverse events (AEs) are easily overlooked. The purpose of this study was to comprehensively evaluate the characteristics of adverse events of fenfluramine.

**Methods:**

The data were extracted from the Food and Drug Administration (FDA) Adverse Event Reporting System (FAERS) database from the third quarter of 2020 to the fourth quarter of 2024 for data cleaning and analysis. To ensure the accuracy and reliability of the study, adverse events of fenfluramine were analyzed using the Reporting Odds Ratio (ROR), Bayesian Confidence Propagation Neural Network (BCPNN), Proportional Reporting Ratio (PRR), and Multi-Item Gamma Poisson Shrinker (MGPS) methods.

**Results:**

Following data deduplication and screening, a total of 9,868 fenfluramine-related adverse event reports were included in this study. The analysis showed that fenfluramine-induced AEs occurred across 24 system organ classes (SOCs). In addition to the typical side effects such as seizure, somnolence, lethargy, status epilepticus, balance disorder and sedation, it is important to pay attention to emerging risks such as pericardial effusion, crying, pneumonia, oxygen saturation decreased, muscle twitching, insomnia, aggression, agitation, mood swings, urinary retention and aortic dilatation. It is notable that aortic valve incompetence and epilepsy are more likely to occur in males and females are more prone to encountering nervous system adverse reactions after fenfluramine treatment. LGS had higher risk after fenfluramine treatment in mitral valve incompetence, constipation, urinary tract infection, fall, lethargy and atonic seizures, while DS had higher risk in pyrexia, illness, nasopharyngitis, influenza, decreased appetite, seizure, generalized tonic-clonic seizure, status epilepticus, myoclonic epilepsy, aggression.

**Conclusion:**

This study provided valuable evidence on the real-world safety of fenfluramine, suggesting that clinicians should place greater emphasis on monitoring its adverse effects during use. Medical staff should pay more attention to cardiac AEs on LGS patients and nervous system AEs on DS patients throughout the entire duration of fenfluramine treatment.

## Highlights

• It is important to pay attention to emerging risks such as pericardial effusion, crying, pneumonia, oxygen saturation decreased, muscle twitching, insomnia, aggression, agitation, mood swings, urinary retention and aortic dilatation.
• It is notable that aortic valve incompetence and epilepsy are more likely to occur in males and females are more prone to encountering nervous system adverse reactions after fenfluramine treatment.
• DS patients are more likely to experience AEs related to the neurological system, whereas LGS patients are more likely to experience cardiac AEs following fenfluramine medication.

## 1 Introduction

Lennox-Gastaut syndrome (LGS) is a rare developmental and epileptic encephalopathy characterized by persistent seizures, cognitive impairment, and aberrant electroencephalogram findings containing delayed spike-wave complexes (Knowles et al., 2024; Nelson and Knupp, 2023). The LGS incidence percentage varied from 14.49 to 28 per 100,000 people (Sullivan et al., 2024). The majority of people with LGS have permanent cognitive impairments and have drop seizures, particularly generalized tonic-clonic seizures (GTCS), which are a major risk factor for sudden unexpected death in epilepsy (Reaven et al., 2018; Knupp et al., 2023). It often appears before the age of eight, but symptoms persist throughout adulthood and need lifetime care with a severe clinical burden. In the United States, half of pediatric epileptic healthcare expenditures are related to LGS, which affects 5% of children with epilepsy (Piña-Garza et al., 2017).

LGS is one of the most difficult epileptic encephalopathies to treat, and the majority of patients are resistant to multiple antiseizure medications. A distinct mode of action is provided by fenfluramine (Fintepla), which has been shown to dramatically lower the frequency of drop seizures (Knupp et al., 2022). It is an oral anti-seizure drug (ASM) with a new mode of action that combines serotonergic activity with positive allosteric modulation effects on sigma-1 receptors (Frampton, 2023). An interim analysis of an open-label extension trial found that individuals with LGS exhibited consistent decreases in drop seizure frequency on fenfluramine therapy, with a particularly strong reduction in GTCS frequency (Knupp et al., 2023). Fenfluramine was given permission as an add-on therapy for seizures caused by Dravet syndrome (DS) in the United States, European Union, and the United Kingdom in 2020 (Knupp et al., 2022). A long-term real-world analysis showed that fenfluramine is well tolerated and reduced the polytherapy load, improving management of DS and relieving caregiver burden (Boncristiano et al., 2025). Fintepla was given approval in the United States via drug repurposing in March 2022 for the treatment of LGS seizures as an add-on therapy to other anti-epileptic medications in patients aged two and above. The FDA has issued boxed warnings for fenfluramine, which has been linked to significant cardiovascular adverse events (AEs) including valvular heart disease (VHD) and pulmonary arterial hypertension (PAH) (Frampton, 2023). Because of these concerns, patients must have cardiac monitoring with echocardiograms before to therapy, every 6 months throughout treatment, and 3–6 months after treatment is completed. If the echocardiography reveals VHD, PAH, or other cardiac problems, healthcare practitioners must weigh the risks and advantages of continuing the patient’s Fintepla medication. The reutilized fenfluramine exerts its anti-epileptic effect through different mechanisms, providing a new treatment approach to the current problem of drug resistance faced by anti-seizure medications (Dini et al., 2023). Compared to its previous usage as an appetite suppressant, it is administered at comparatively lower doses in the treatment of DS and LGS (Dini et al., 2023). This is why we cannot simply copy the previous experience in medication. Further research is needed to look into the possible AE signals of fenfluramine in real-world scenarios, identify rare and severe AEs associated with this medication, and promote the safe use of Fintepla among LGS and DS patients.

Drug repurposing is an emerging technique of reassigning existing pre-approved medicines for new purposes, potentially resulting in cheaper overall development costs and faster development schedules for rare illnesses (Pushpakom et al., 2019). Fenfluramine, an ancient medicine with a new use in uncommon disorders, may have undiscovered adverse events. There is limited data on LGS and DS populations from real-world clinical settings, possibly because analyzing individuals with uncommon illnesses is frequently accompanied with sample size issues. Due to clinical trial limits, certain delayed and uncommon adverse events may be undetected. The FDA Adverse Event Reporting System (FAERS) collects substantial drug safety signal data and is a sophisticated tool for assessing adverse drug-related events. The goal of this study was to look at fenfluramine-related adverse events and patient characteristics in the FAERS database. The findings might be a valuable resource for future clinical trials and enhance medication safety for persons with LGS.

## 2 Materials and methods

### 2.1 Data source and collection

We performed a retrospective pharmacovigilance analysis on fenfluramine adverse events (AEs) using the FAERS database, a publicly accessible database of safety reports filed by pharmaceutical firms, pharmacists, and consumers worldwide since 2004. It is the largest spontaneous reporting system database in the world, containing more than 9 million individual drug-related adverse event reports that have been submitted by consumers, healthcare professionals, doctors, pharmacists, and industry professionals (Liu et al., 2024). The FDA launched Fintepla in June 2020, and AEs were gathered from the third quarter of 2020 to the fourth quarter of 2024 (Wirrell et al., 2022).

### 2.2 Data processing

**FIGURE 1:**
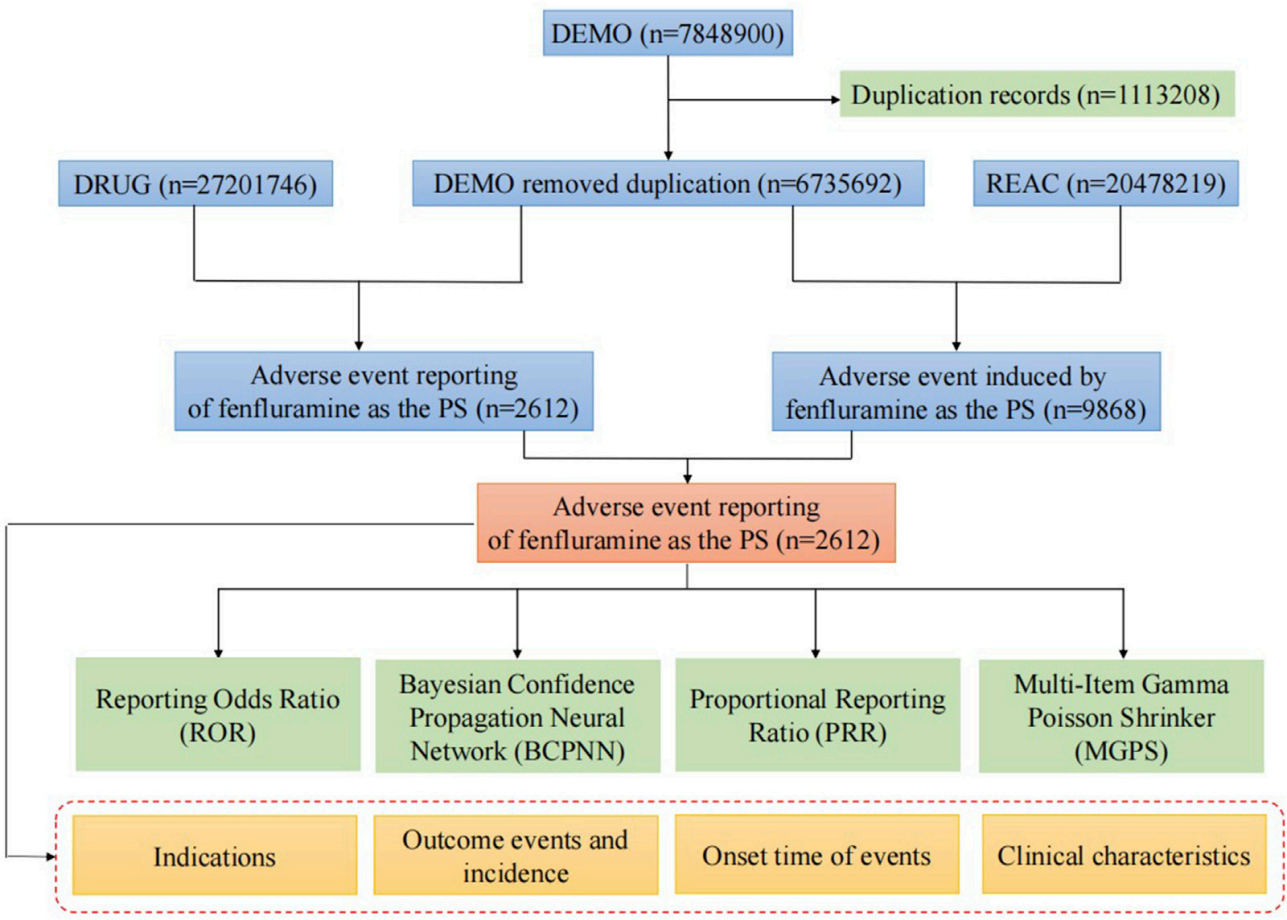
Flow diagram of the study (DEMO, demographic and administrative information; DRUG, drug Information; REAC, preferred terminology for adverse drug reactions; PS, primary suspect drug).

The FAERS data were acquired from the Quarterly Data Extract Files, which are publicly accessible at https://fis.fda.gov/extensions/FPD-QDE-FAERS/FPD-QDE-FAERS.html. We obtain the FAERS data and clean the data via RStudio following the instructions from the FDA. We searched the whole fenfluramine drug nomenclature, including trade names, generic names, non-proprietary names, and medicine brands, using the Medical Subject Headings thesaurus (https://www.ncbi.nlm.nih.gov/mesh). To find similar reports, the commercial name “Fintepla” and generic name “fenfluramine” were utilized. The following brand or generic names of the medications were filtered out of the database by the study: “fenfluramine,” “Fintepla,” “fenfluramine hydrochloride,” and “Pondimin.” Only the reports of fenfluramine with role code as the primary suspected drug were chosen for analysis. When referring to the names of AEs in the reports, preferred terms (PTs) from the Medical Dictionary for Regulatory Activities (MedDRA) should be used for consistent encoding. The study included all PTs that fell within the larger category of diseases and infestations known as system-organ classes (SOC). We keep the report with the highest FDA_DT value for reports with the same CASEID. We keep the report with the highest PRIMARYID value when the CASEID and FDA_DT are same. A final dataset that is prepared for analysis was created by compiling the cleaned and standardized data. Figure 1 depicts the comprehensive screening procedure. In keeping with the emphasis of our analysis, this dataset only contained cases in which fenfluramine was identified as the primary suspected drug (PS). Every fenfluramine adverse event report was examined at the System Organ Class (SOC) and PT levels. In this study, we categorized AE outcomes of fenfluramine into two groups: severe AEs and non-severe AEs. The severe AEs group encompasses occurrences of death, life-threatening situations, hospitalization or prolonged hospital stay, permanent or severe disability or impairment, congenital anomalies, or other serious medical event. If none of the above AEs occurred, they were included in the non-serious AEs group. Additionally, when a single report contained multiple AEs, if any of the above serious AEs occurred, they were included in the serious AEs group.

### 2.3 Statistical analysis

Disproportionality analysis is a useful tool for identifying and detecting drug-related adverse reaction signals in pharmacovigilance studies (van Puijenbroek et al., 2002), and methods with high sensitivity can identify more potential AEs and reduce the likelihood of missing true signals, while methods with high specificity can reduce the proportion of false positive signals (Jiao et al., 2024). To increase the reliability of the results, we used various disproportionality analysis techniques to reduce the bias of false-positive results caused by one method, such as reporting odds ratio (ROR) and proportional reporting ratio (PRR), Bayesian confidence propagation neural network (BCPNN), and multi-item gamma Poisson shrinker (MGPS). A preferred terminology is considered a positive signal if it simultaneously meets the thresholds of all four algorithms, and the equations and criteria for the four algorithms are detailed in Table 1. Data were analyzed using Microsoft Excel 2021 and R 4.3.0.

**TABLE 1:** Calculation formula and standard of signal detection.

Algorithm	Calculation formula	Criterion
ROR	$\text{ROR}=\frac{a/c}{b/d}=\frac{ad}{bc}$	a ≥ 3
	$95\%\text{CI}=e^{\ln\left(ROR\right)\pm 1.96\sqrt{\frac{1}{a}+\frac{1}{b}+\frac{1}{c}+\frac{1}{d}}}$	95%CI (lower limit) > 1
PRR	$\text{PRR}=\frac{a/\left(a+b\right)}{c/\left(c+d\right)}$	a ≥ 3, PRR ≥ 2
	$\chi^{2}=\frac{{\left(ad-bc\right)}^{2}\left(a+b+c+d\right)}{\left(a+b\right)\left(a+c\right)\left(c+d\right)\left(b+d\right)}$	χ^2^ ≥ 4
EBGM	$\text{EGBM}=\frac{a\left(a+b+c+d\right)}{\left(a+b\right)\left(a+c\right)}$	EBGM05 > 2
	$\text{EBGM}05=e^{\ln\left(EBGM\right)-1.64\sqrt{\frac{1}{a}+\frac{1}{b}+\frac{1}{c}+\frac{1}{d}}}$	
BCPNN	$\text{IC}=\log_{2}\frac{a\left(a+b+c+d\right)}{\left(a+b\right)\left(a+c\right)}$	a ≥ 3
	$E\left(IC\right)=\log_{2}\frac{\left(a+\gamma_{11}\right)\left(N+\alpha \right)\left(N+\beta \right)}{\left(N+\gamma \right)\left(a+b+\alpha_{1}\right)\left(a+c+\beta_{1}\right)}$ $V\left(IC\right)=\frac{1}{{\left(\ln2\right)}^{2}}\left[\frac{N-a+\gamma -\gamma_{11}}{\left(a+\gamma_{11}\right)\left(N+1+\gamma \right)}+\frac{N-a-b+\alpha -\alpha_{1}}{\left(a+b+\alpha_{1}\right)\left(N+1+\alpha \right)}+\frac{N-a-c+\beta -\beta_{1}}{\left(a+c+\beta_{1}\right)\left(N+1+\beta \right)}\right]$ $\gamma =\gamma_{11}\frac{\left(N+\alpha \right)\left(N+\beta \right)}{\left(a+b+\alpha_{1}\right)\left(a+c+\beta_{1}\right)}$ $95\%CI=E\left(IC\right)\pm 1.96\sqrt{V\left(IC\right)}$ Where α = α_1_+α_2_, β = β_1_+β_2_, N = a+b+c+d, and the value of α_1_, α_2_, β_1_, β_2_ and γ_11_ were defined as 1	The lower limit of 95%CI (IC025) > 0

Abbreviations:a, number of reports containing both the target drug and target adverse drug reaction; b, number of reports containing other adverse drug reaction of the target drug; c, number of reports containing the target adverse drug reaction of other drugs; d, number of reports containing other drugs and other adverse drug reactions. 95%CI, 95% confidence interval; χ^2^, chi-squared; IC, information component; IC025, the lower limit of 95% CI, of the IC; EBGM05, the lower limit of 90% CI, of the EBGM.

### 2.4 Time to onset analysis

The gap between EVENT_DT (the date of ADE onset in the DEMO file) and START_DT (the date of medication commencement in the THER file) was used to calculate the time to onset (TTO) of fenfluramine-related ADEs. Excluded were cases with mistakes (not specific to a day, month, or year) or missing dates (either the start of AEs or the start of treatment). Furthermore, because this would provide a negative time-to-onset computation, instances where the starting date of AEs occurred prior to the start date of fenfluramine medication were also eliminated (Liu et al., 2024). In this work, we measured TTO features using the median, quartile, minimum, maximum, and Weibull shape parameter (Kinoshita et al., 2020).

## 3 Results

### 3.1 General characteristics

From the third quarter of 2020 to the fourth quarter of 2024, the FAERS database received a total of 7,848,900 reports. Following data deduplication and screening, 9,868 adverse reaction reports involving 2,612 patients were identified, with fenfluramine designated as the PS drug.

Clinical characteristics of AEs to fenfluramine are shown in Table 2. In terms of gender, approximately 44.5% patients were female and 49.6% were male. In addition to the majority of unknown ages, the vast majority of reports focused on the age group of 0–17 years (49.0%), followed by the age group of 18–64 years (22.7%). In terms of reporting sources, the vast majority of reports were provided by consumer (60.6%). The top country reported was the United States (85.6%). Excluding unknown outcome, other serious (important medical event) get the most reports (38.9%). Serious AEs accounted for 66.2%. Since its launch in 2020, the number of reported AEs has shown a steady annual increase, peaking in 2024 with 43.5% of the total reports.

**TABLE 2:** Characteristics of reports associated with fenfluramine from the FAERS database (Q3 2020-Q4 2024).

Factors	Number of events (%)
Case reports	2612
Gender	
Female	1163 (44.5)
Male	1295 (49.6)
Unknown	154 (5.9)
Age (year)	
<18	1279 (49.0)
18–64	592 (22.7)
>65	3 (0.1)
Unknown	738 (28.3)
Reporter	
Consumer	1584 (60.6)
Health Professional	161 (6.2)
Physician	732 (28.0)
Pharmacist	60 (2.3)
Unknown	75 (2.9)
Reporter country	
United States	2237 (85.6)
Japan	127 (4.9)
Germany	53 (2.0)
Others	195 (7.5)
Outcome	
Hospitalization (initial or prolonged)	626 (24.0)
Death	60 (2.3)
Congenital Anomaly	4 (0.2)
Life threatening	17 (0.7)
Disability	5 (0.2)
Other serious (important medical event)	1017 (38.9)
Unknown	883 (33.8)
Seriousness	
Serious	1729 (66.2)
Non-serious	883 (33.8)
Reporting year	
2020	24 (0.9)
2021	378 (14.5)
2022	525 (20.1)
2023	550 (21.1)
2024	1135 (43.5)

### 3.2 Signal detection of fenfluramine at the system organ class (SOC) level

The signal strength of fenfluramine at the SOC level is shown at Table 3. After conducting an analysis, we have identified a total of 24 organ systems that are affected by adverse drug reactions caused by fenfluramine. The most frequently reported SOC was nervous system disorders. Ranked by the number of reported cases, the top three SOCs were nervous system disorders (n = 2497, 26.6%), General disorders and administration site conditions (n = 1188, 12.7%), and psychiatric disorders (n = 832, 8.9%). Nervous system disorders, cardiac disorders and metabolism and nutrition disorders demonstrated a strong positive signal across all four algorithms, aligning with descriptions in the fenfluramine drug label, which suggests high data reliability.

**TABLE 3:** The signal strength of fenfluramine at the System Organ Class (SOC) level.

System organ class (SOC)	Case number	ROR (95% CI)	PRR (χ^2^)	EBGM (EBGM05)	IC (IC025)
Nervous system disorders	2497	4.4 (4.2–4.6)	3.54 (4887.32)	3.53 (3.4)	1.82 (1.76)
General disorders and administration site conditions	1188	0.64 (0.6–0.68)	0.68 (214.99)	0.68 (0.65)	−0.55 (−0.64)
Psychiatric disorders	832	1.62 (1.51–1.74)	1.57 (181.77)	1.57 (1.48)	0.65 (0.55)
Injury, poisoning and procedural complications	826	0.62 (0.58–0.67)	0.65 (176.77)	0.65 (0.61)	−0.62 (−0.72)
Cardiac disorders	779	4.39 (4.08–4.73)	4.12 (1875.91)	4.12 (3.87)	2.04 (1.93)
Infections and infestations	740	1.35 (1.25–1.45)	1.32 (61)	1.32 (1.24)	0.4 (0.29)
Investigations	636	1.11 (1.03–1.21)	1.11 (6.97)	1.11 (1.03)	0.15 (0.03)
Gastrointestinal disorders	531	0.67 (0.62–0.73)	0.69 (80.56)	0.69 (0.64)	−0.54 (−0.66)
Metabolism and nutrition disorders	448	2.45 (2.23–2.7)	2.39 (368.01)	2.39 (2.2)	1.25 (1.12)
Respiratory, thoracic and mediastinal disorders	307	0.68 (0.61–0.77)	0.69 (43.57)	0.69 (0.63)	−0.53 (−0.7)
Skin and subcutaneous tissue disorders	118	0.22 (0.19–0.27)	0.23 (316.8)	0.23 (0.2)	−2.11 (−2.37)
Vascular disorders	112	0.62 (0.51–0.74)	0.62 (26.46)	0.62 (0.53)	−0.69 (−0.96)
Musculoskeletal and connective tissue disorders	99	0.19 (0.15–0.23)	0.2 (344.21)	0.2 (0.17)	−2.35 (−2.64)
Renal and urinary disorders	78	0.44 (0.35–0.55)	0.45 (54.65)	0.45 (0.37)	−1.16 (−1.49)
Eye disorders	61	0.31 (0.24–0.4)	0.32 (92.45)	0.32 (0.26)	−1.66 (−2.03)
Congenital, familial and genetic disorders	35	1.36 (0.98–1.9)	1.36 (3.34)	1.36 (1.03)	0.44 (−0.04)
Blood and lymphatic system disorders	24	0.14 (0.09–0.21)	0.14 (128.95)	0.14 (0.1)	−2.84 (−3.41)
Immune system disorders	22	0.2 (0.13–0.3)	0.2 (72.27)	0.2 (0.14)	−2.34 (−2.94)
Reproductive system and breast disorders	16	0.28 (0.17–0.45)	0.28 (29.83)	0.28 (0.19)	−1.84 (−2.54)
Hepatobiliary disorders	15	0.18 (0.11–0.3)	0.18 (55.13)	0.18 (0.12)	−2.45 (−3.17)
Endocrine disorders	10	0.37 (0.2–0.7)	0.38 (10.41)	0.38 (0.22)	−1.41 (−2.28)
Ear and labyrinth disorders	7	0.18 (0.08–0.37)	0.18 (27.01)	0.18 (0.09)	−2.5 (−3.52)
Neoplasms benign, malignant and unspecified (incl cysts and polyps)	6	0.01 (0.01–0.03)	0.02 (401.43)	0.02 (0.01)	−6.05 (−7.14)
Pregnancy, puerperium and perinatal conditions	3	0.09 (0.03–0.29)	0.09 (26.18)	0.09 (0.04)	−3.41 (−4.85)

### 3.3 Signal detection of fenfluramine at the preferred terms (PT) level

**FIGURE 2:**
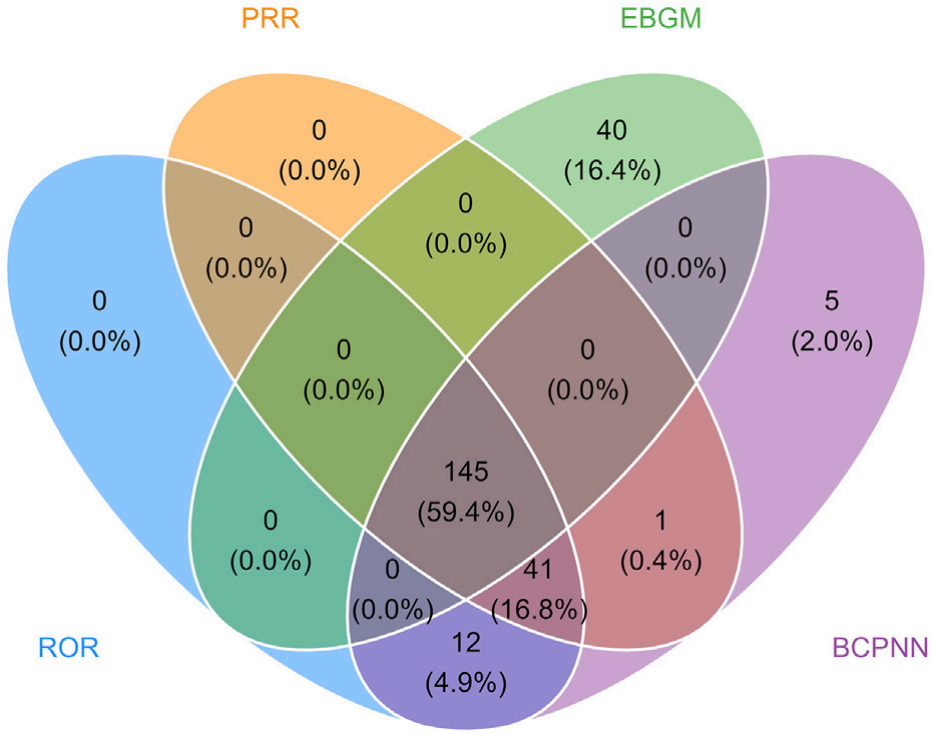
Venn diagram of preferred term (PT) signals meeting the criteria of four algorithms.

**FIGURE 3:**
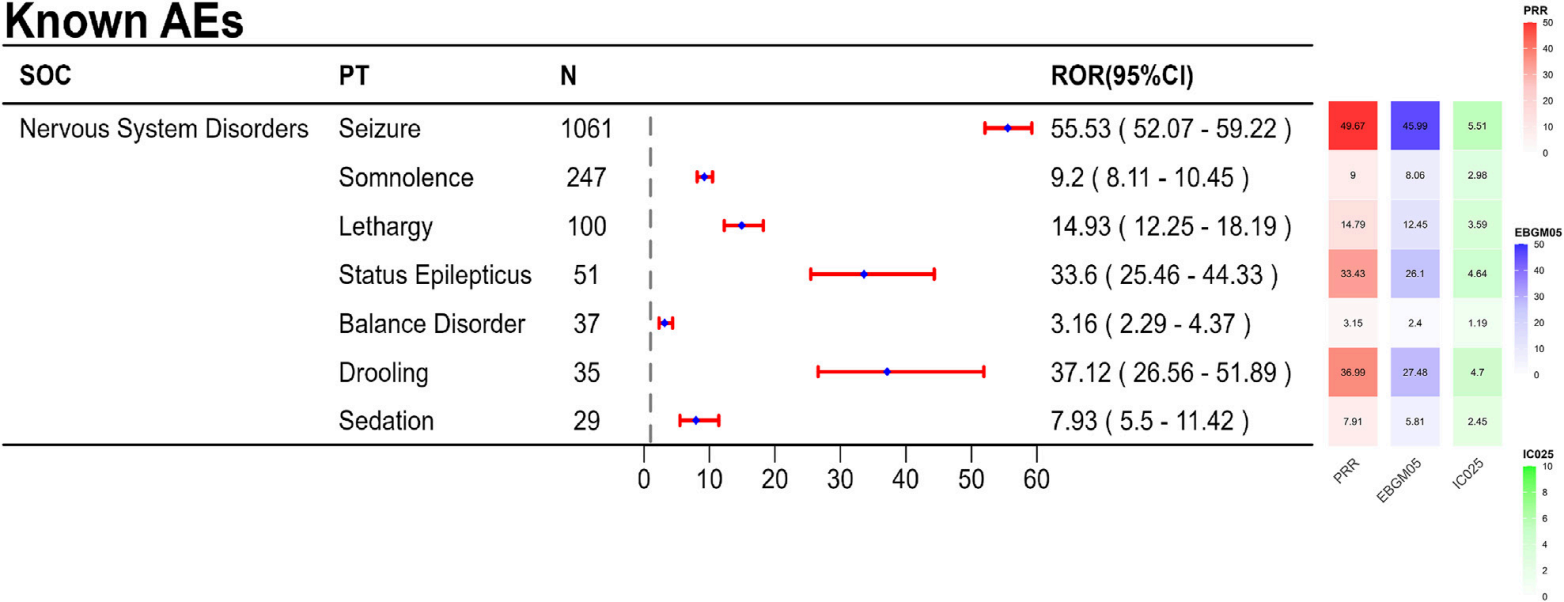
A forest plot and heat map of known AEs meeting the criteria of four algorithms.

**FIGURE 4:**
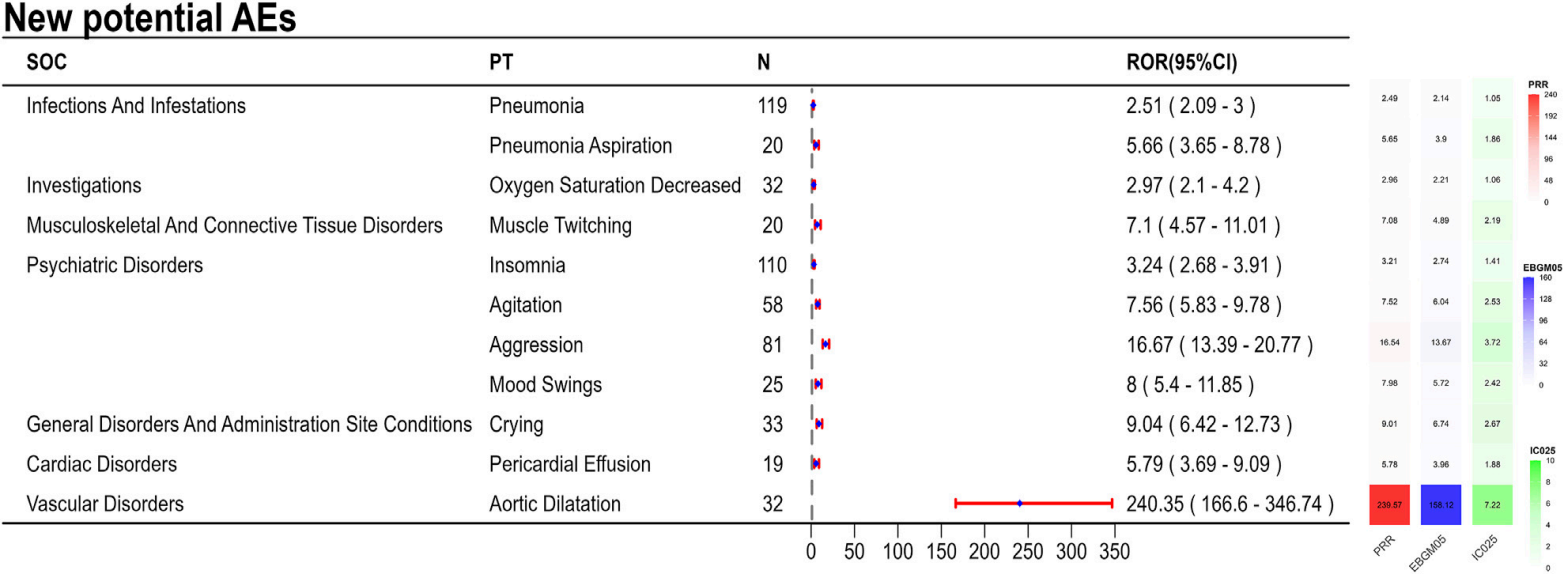
A forest plot and heat map of new potential AEs meeting the criteria of four algorithms.

The final results showed that 145 PTs met the positive criteria across all four algorithms. The top 50 AEs associated with fenfluramine at the PT level is shown at Supplementary Table S1. The Venn diagram visually illustrated the AEs that met the positive threshold of all four algorithms at the PT level in Figure 2. Among the top 50 most common AEs, several events were identified that aligned with those listed on the drug label, including weight decreased, echocardiogram abnormal, decreased appetite, seizure, somnolence, lethargy, status epilepticus, balance disorder, drooling, sedation, abnormal behavior and irritability. Valvular heart disease (such as tricuspid valve incompetence, mitral valve incompetence, aortic valve incompetence, pulmonary valve incompetence and mitral valve thickening) and pulmonary arterial hypertension are clearly indicated in the black box warning. A forest plot and heat map of known AEs meeting the criteria of four algorithms was shown in Figure 3. Additionally, several noteworthy AEs not included on the drug label were identified, such as pericardial effusion, crying, pneumonia, oxygen saturation decreased, muscle twitching, insomnia, aggression, agitation, mood swings, urinary retention and aortic dilatation. A forest plot and heat map of new potential AEs meeting the criteria of four algorithms was shown in Figure 4.

### 3.4 Sensitivity analysis

60.6% of the reports were provided by consumers. Due to their lack of professional medical knowledge, this might affect the reliability of certain signals. In order to verify the reliability of the new potential AEs we have discovered, we conducted a sensitivity analysis with the reporting sources limited to medical staffs, including health professional, physician and pharmacist. In Supplementary Table S2, we found that aortic dilatation, insomnia, agitation, aggression, pericardial effusion, crying, muscle twitching and mood swings still maintain a positive signal. This indicates the robustness of our results.

### 3.5 Time-to-onset (TTO) analysis

**FIGURE 5:**
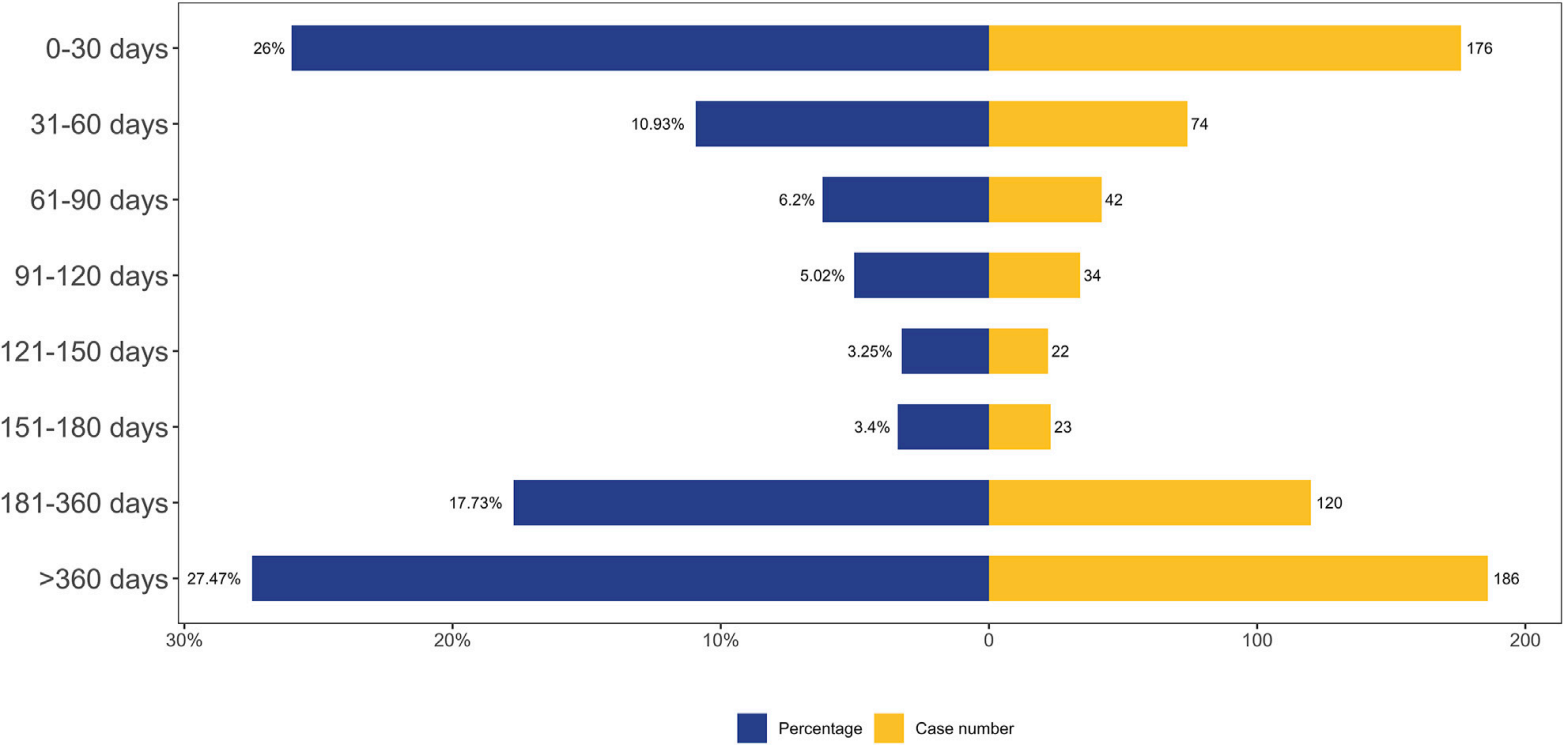
TTO analysis of fenfluramine-related AEs counted in days.

A total of 677 AEs were associated with effective TTO reports. The distribution of onset times for these AEs is shown in Figure 5. The median of TTO was determined as 141 days and the interquartile range (IQR) was 29–397 days. As shown in Figure 5, most cases occurred more than 1 year (n = 186, 27.47%) of fenfluramine administration, followed by the first month (n = 176, 26.00%). The number of ADEs decreased over time within 150 days, with 74 AEs (10.93%) occurring in the second month and 42 ADEs (6.20%) in the third month. AEs were least likely to occur during the fifth to sixth month of treatment, with rates of 3.25% and 3.40% respectively, but significantly rose afterwards. Notably, our data revealed that a considerable 27.47% of AEs remained possible following a year of fenfluramine treatment. These findings emphasize the importance of monitoring patients for potential AEs throughout the course of fenfluramine therapy, even beyond the initial months.

We performed Weibull distribution tests on both the whole patient population in Table 4 and the fenfluramine-associated adverse events to see if the risk of these events rises or falls with time. An early failure-type curve is thought to indicate a decreasing likelihood of negative effects with time when the form parameter β is less than 1 and its 95% confidence interval (CI) is likewise below 1 (Mazhar et al., 2021). The Weibull distribution test for TTO indicated that the upper limit of the 95% CI for the shape parameter (β) was 0.74 (less than 1), suggesting that the probability of AEs gradually decreased over time.

**TABLE 4:** Weibull distribution tests on TTO analysis.

Cases	TTO (days)		Weibull distribution				Failure type
			Scale parameter		Shape parameter		
	Media (IQR)	Min-Max	α	95% CI	β	95% CI	
677	141 (29–397)	1–7412	220.35	195.28–245.43	0.70	0.66–0.74	Early failure

### 3.6 Gender-based difference in risk signals for fenfluramine

**FIGURE 6:**
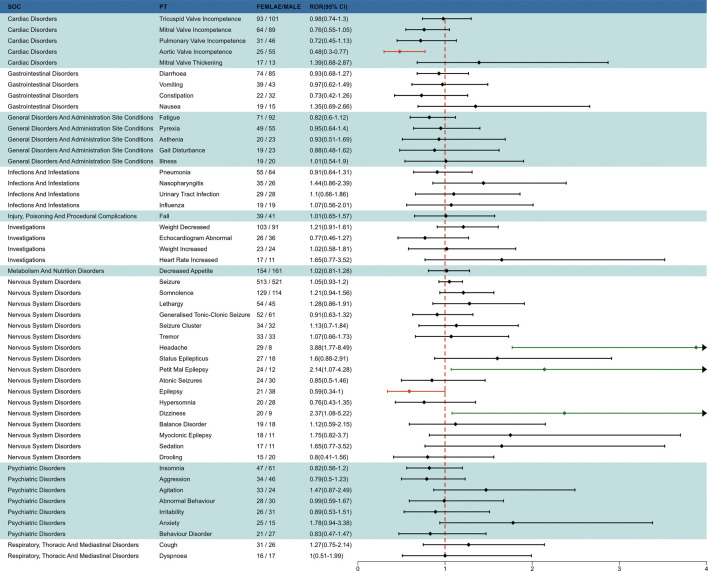
Subgroup analysis of gender-differentiated risk signals in fenfluramine.

To investigate the effect of gender factors on AEs of fenfluramine, we used the ROR method to identify 50 PTs with disproportionate AE incidence between males and females, categorized by SOC. The results are presented in Figure 6. Some AEs such as headache, petit mal epilepsy and dizziness were more common in females, while aortic valve incompetence and epilepsy were more common in males.

### 3.7 Indication-based difference in risk signals for fenfluramine

**FIGURE 7:**
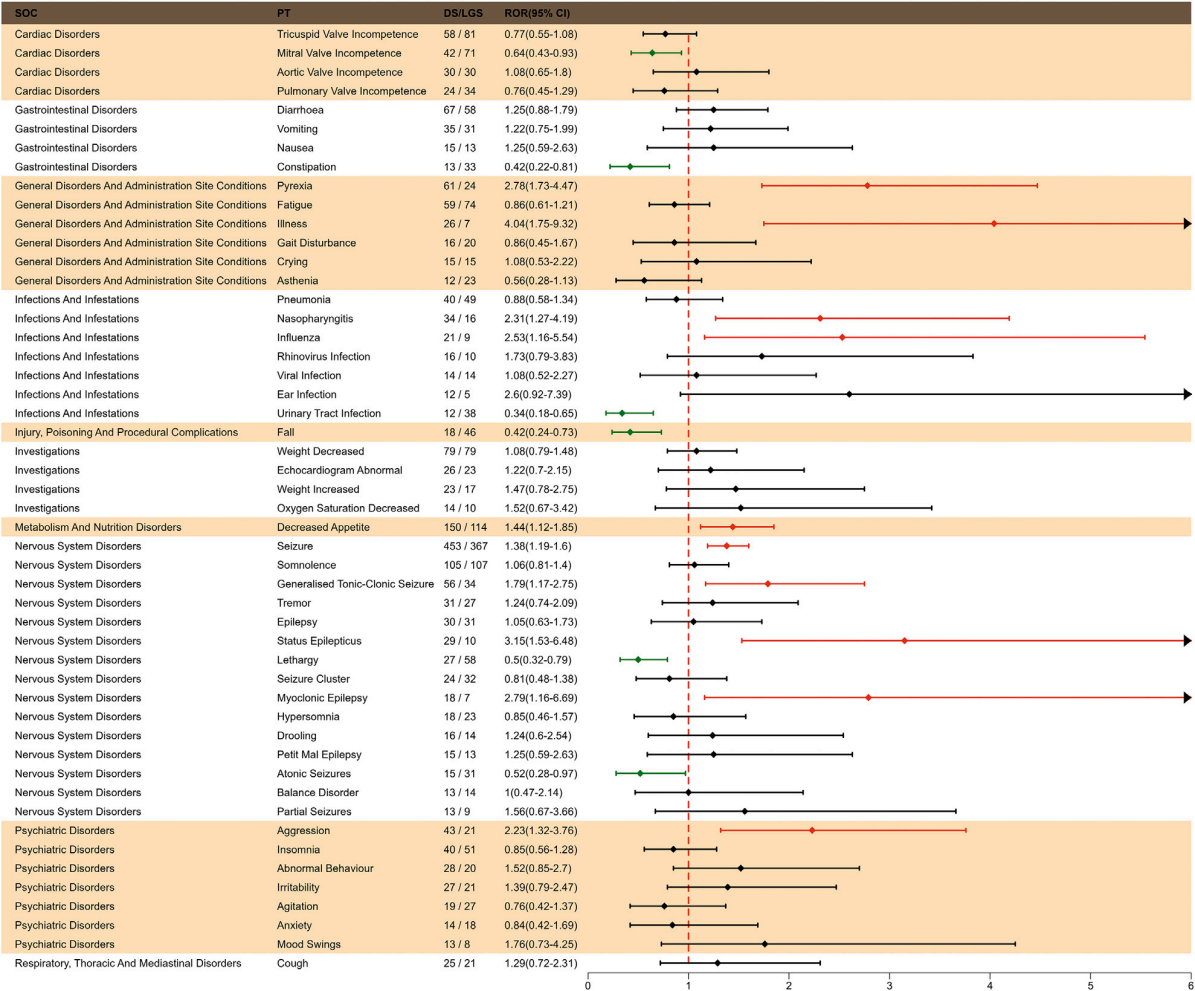
Subgroup analysis of indication-differentiated risk signals in fenfluramine.

To investigate the effect of indication factors on AEs of fenfluramine, we performed an indication subgroup analysis between LGS and DS in top 50 frequency of adverse events at the PT level. We found that LGS had higher risk in mitral valve incompetence, constipation, urinary tract infection, fall, lethargy and atonic seizures, while DS had higher risk in pyrexia, illness, nasopharyngitis, influenza, decreased appetite, seizure, generalized tonic-clonic seizure, status epilepticus, myoclonic epilepsy, aggression (Figure 7).

## 4 Discussion

Fenfluramine, an antiseizure medication (ASM) with serotonergic and sigma-1 receptor activity, is used to treat seizures in individuals with LGS and DS. It is a racemic combination of D- and L-enantiomers and a derivative of amphetamine (Fenfluramine, 2022). The cause of epileptic seizures is an imbalance between excitatory (glutamatergic) and inhibitory (e.g., γ-aminobutyric acid-ergic, or GABAergic) input from neurons. Furthermore, by activating 5-HT2A and 5-HT2C receptors and releasing 5-HT at GABAergic synapses, fenfluramine improves GABAergic neurotransmission (Guiard and Di Giovanni, 2015). By regulating the sigma-1 interaction with the N-methyl-D-aspartic acid receptor, which dampens calcium influx and reduces seizure activity at glutaminergic synapses, fenfluramine recovers the loss of GABAergic tone (Martin et al., 2021). In addition to restoring dendritic arborization and reducing myelin degradation and neuroinflammation, fenfluramine has been demonstrated to have antiseizure effects, including a global tonic-clonic decrease, which may indicate disease-modifying effects (Tiraboschi et al., 2020). Fenfluramine demonstrated persistent, clinically relevant monthly drop seizure frequency reductions for more than a year in the ongoing open-label extension research, according to a randomized, double-blind, placebo-controlled, international, phase III trial (Knupp et al., 2023). However, more clinical trials and research are required to thoroughly evaluate fenfluramine’s efficacy and safety, guaranteeing its effectiveness and dependability in practical usage, given that it is linked to specific adverse responses in clinical applications and trial data is still few. This study conducted an in-depth analysis of real-world AEs associated with fenfluramine using FAERS database data, with key points discussed below.

### 4.1 Analysis of signal basic information AEs

Our findings show that fenfluramine-related side effects occur similar frequently in males (49.6%) than in females (44.5%). Age at diagnosis for individuals with LGS overall ranged from 2 to 15 years (Sullivan et al., 2024). Across all age ranges of age, the higher proportion of AEs in children (<18 years) may be related to the clinical characteristics of this population. Due to the epidemiological characteristics of LGS and DS, fenfluramine is mainly used for children in other clinical studies. In the drug instructions, fenfluramine is indicated for the treatment of seizures associated with DS and LGS in patients 2 years of age and older. It should be noted that the AE reports included in this study not only involve children (49.0%), but also concern adults (22.8%). The primary reporters were consumers (60.6%) and physicians (28.0%). Adverse drug reactions were valued by both consumers and health professionals, indicating a high patient focus on drug safety and possibly reflecting high expectations for the treatment of LGS and DS. Raising awareness and promoting treatment adherence may be achieved by educating patients and their families, particularly about the possible dangers associated with the medication.

### 4.2 Known AEs

In this investigation, there were 145 AEs with positive signals, primarily related to illnesses of the neurological system, psychiatry, heart, and metabolism, and they were typically in line with the description on the prescription insert. The present study found that the AEs associated with fenfluramine were mainly centered on nervous system disorders, which is generally consistent with the prescribing information of fenfluramine, such as seizure, somnolence, lethargy, status epilepticus, balance disorder and sedation. Reduced hunger and somnolence were the most frequent AEs in a multicenter, double-blind, placebo-controlled, parallel-group randomized clinical trial (Knupp et al., 2022). The AEs that were substantially linked to fenfluramine in the entire analysis were tiredness, weight loss, diarrhea, and reduced appetite, according to a systematic review and meta-analysis (Tabaee Damavandi et al., 2023). These common AEs can also be observed in the previous clinical trials of fenfluramine for treating pharmacoresistant epilepsies. An open-label extension study for DS patients showed that the main adverse effects related to the use of fenfluramine were decreased appetite, fatigue, diarrhea, and pyrexia (Dini et al., 2022). In a phase II, open-label study (NCT02655198), the most common AE was decreased appetite for LGS patients (Dini et al., 2022). Although diarrhea is not visible in Supplementary Table S1, it exhibited a positive signal in the ROR and BCPNN algorithms. In our investigation, we found all of these adverse events.

Both direct and indirect serotonergic actions are demonstrated by fenfluramine. By interfering with the transmitter’s vesicular storage and preventing its pre-synaptic re-uptake, this serotonin-releasing drug raises extracellular serotonin (5-HT) levels. In order to suppress appetite, fenfluramine potentiates serotonergic transmission by stimulating 5-HT1B, 5-HT2C, and possibly 5-HT2B receptors. This is accomplished indirectly through interaction with the serotonin transporter (SERT), which inhibits 5-HT uptake and stimulates 5-HT release (Halford et al., 2011). The hypothalamic melanocortin system’s proopiomelanocortin (POMC) neurons, which control feeding and energy homeostasis, are then stimulated by 5-HT via 5-HT2C receptors (He et al., 2021).

Though there have been no reports of VHD or PAH in the clinical program thus far, it is noteworthy that fenfluramine is linked to a risk of valvular heart disease and pulmonary arterial hypertension (VHD/PAH) because of cases that happened when it was promoted as an anorectic medication (Wirrell et al., 2024). Excessive serotonergic activation of the damaged heart valve tissue causes fenfluramine-induced valvulopathy (Rajamannan et al., 2001). There is evidence to suggest that distinct serotonergic pathways mediate appetite suppression and cardiac valvulopathy (Odi et al., 2021). The leaflets of the aortic and mitral valves (as well as the pulmonary arteries) express serotonin 5HT2B receptors. The development of valvular heart disease may be explained by the activation of these cells by 5HT2B agonist medications, which may lead to collagen production and fibroblast proliferation (Andrejak and Tribouilloy, 2013). PAH can be caused by fenfluramine’s induction of pulmonary artery smooth muscle cell proliferation through serotonergic overactivity (Adnot et al., 2013). The Risk Evaluation and Mitigation Strategy program in the US and the Controlled Access Program in the European Union both impose restrictions on the distribution of fenfluramine. In order to detect any cardiac anomalies before a patient develops symptoms or advances to VHD or PAH, these programs necessitate routine echocardiography monitoring. Experts concur that there are more advantages to using fenfluramine than possible cardiac risks (Wirrell et al., 2024).

### 4.3 New potential AEs

Since DS and LGS are rare diseases, the number of cases involved in clinical practice is relatively small, which makes some rare AEs difficult to be detected. In a clinical practice, only one patient among the 4 cases reported an AE (weight loss) secondary to treatment with fenfluramine (Wirrell et al., 2024). In an open-label extension study, authors said that 22.3% of the patient population withdrew from the study for lack of efficacy, which may lead to an underreporting of AEs over time (Knupp et al., 2023). Compared with the recent reviews and controlled trials, this study included 9,868 AE reports from 2,612 patients, which gives us an advantage in identifying new potential AEs. During fenfluramine medication, this investigation found a number of novel possible AE signals, such as pericardial effusion, weeping, pneumonia, reduced oxygen saturation, muscle twitching, sleeplessness, hostility, agitation, mood swings, urine retention, and aortic dilatation. Despite the comparatively small number of reported psychiatric symptoms, the strong signal intensity of these symptoms suggested a possible link. The oxytocin and vasopressin levels that are generated by fenfluramine may be linked to the development of mental symptoms. In the rat brain, dextro-fenfluramine selectively activates vasopressinergic and oxytocinergic neurons (Mikkelsen et al., 1999). Emotions and actions, including social behavior and anxiety, are controlled by the ratio of oxytocin to vasopressin (Heinrichs et al., 2009). Drug-induced pulmonary hypertension may be associated with pericardial effusion. Fluid accumulation in the pericardial area, inadequate reabsorbing system drainage, and varying degrees of pericardial effusion are all possible side effects of PAH (Ebrahimi et al., 2025). Aortic dilatation may result from the potentiation of transforming growth factor-beta (TGF-β) levels caused by dexfenfluramine therapy (Launay et al., 2002). Defective vascular matrix homeostasis involving TGF-β dysregulation is assumed to be the cause of the gradual degradation of the aortic media, which leads to vessel dilatation (Rueda-Martínez et al., 2017). The serotoninergic pathways may be the explanation behind fenfluramine’s increased risk of urine retention. By enhancing sympathetic activity and suppressing parasympathetic activity, serotoninergic pathway activation encourages urine storage (Crisafulli et al., 2022).

### 4.4 TTO analysis

Since it identifies certain risk windows and promotes the avoidance or early identification of adverse responses, the temporal link between administration and time of beginning is essential for evaluating medication safety. According to TTO study, the median time of fenfluramine-related adverse events was 141 days, and the majority of instances (n = 186, 27.47%) happened after more than a year of fenfluramine medication. The results showed how crucial it is to keep a closely monitoring on the AEs that patients encounter throughout the course of their therapy.

### 4.5 Gender-based difference in risk signals for fenfluramine

Females are more likely to experience AEs related to the neurological system, such as headache, dizziness, and petit mal epilepsy, as seen in Figure 6. The fact that there are gender disparities in several central serotonergic system functions may be connected to this (Soloff et al., 2005). Compared to males, women have been shown to have lower pain inhibition, increased pain facilitation, and increased pain sensitivity in lab-based investigations (Racine et al., 2012a; Racine et al., 2012b). Women are more likely than males to suffer from fibromyalgia, migraine, and chronic tension-type headache, among other prevalent chronic pain illnesses (Racine et al., 2012a). It is noteworthy that following fenfluramine medication, males are more prone to experience epilepsy and aortic valve insufficiency. It serves as a warning to exercise caution and warn about the signs of aortic valve incompetence, particularly in male patients who present with such symptoms while using the medication, even if prior clinical studies did not disclose any drug-related major cardiovascular adverse events. The gender difference in drug-induced epilepsy may be related to the difference in hormone levels between men and women. By varyingly altering neuronal excitability through both genetic and non-genomic pathways, endogenous steroid sex hormones (especially estrogen and progesterone) play a significant role in sex variations in epilepsy. Animal models have demonstrated that progesterone has a protective effect against seizures brought on by electrical stimulation or chemoconvulsants, mostly due to its neuroinhibitory impact (Hophing et al., 2022). These results highlight how crucial it is to pay attention to adverse responses in patients of various genders throughout clinical practice. However, it is important to note that further clinical data is needed to confirm these results.

### 4.6 Indication-based difference in risk signals for fenfluramine

DS patients are more likely to experience AEs related to the neurological system, whereas LGS patients are more likely to experience cardiac AEs following fenfluramine medication, as seen in Figure 7. This discovery enables medical practitioners to utilize fenfluramine to monitor adverse responses for patients with various indications in a targeted manner. The accurate identification of uncommon disorders is especially crucial before this. Diagnosis is more difficult in the LGS population due to its greater heterogeneity, which includes a variety of symptoms, several etiologies, no known genetic mutation, and a lack of illness biomarkers (Bourgeois et al., 2014; Asadi-Pooya, 2018). In diagnosing LGS, doctors consistently regarded multiple seizure types, abnormal electroencephalogram patterns and findings, developmental delay, and intellectual disabilities as important distinguishing features. In diagnosing DS, they considered the genetic test for the SCN1A mutation, multiple seizure types, and prolonged seizures, especially febrile seizures, as important distinguishing features (Shah et al., 2025).

Given that the AEs of fenfluramine vary in different indications, doctors need to conduct regular echocardiographic follow-up for LGS patients or perform neurobehavioral monitoring for DS patients. The lives of patients with LGS are affected not only by frequent and disabling seizures but also by a range of comorbidities, including physical disability, cognitive impairment, behavioural problems (such as hyperactivity, aggressiveness, and autistic traits), and sleep disturbances (Auvin et al., 2025). This should include careful consideration of the balance of risk-benefit ratio based on the patient’s specific condition when choosing antiseizure medications treatment. Not only should doctors pay attention to potential drug interactions with medications used to treat comorbidities but also some antiseizure medications may cause or worsen certain comorbidities. Therapeutic drug monitoring is another important measure to enhance the safety of fenfluramine usage, which helps ensure quality of treatment through dose adjustment based on individual drug exposure by determining plasma drug concentrations. A faster method was reported by Federica Pigliasco using a novel liquid chromatography-tandem mass spectrometry method, which allows cannabidiol to be measured in addition to fenfluramine and norfenfluramine, from a smaller volume of plasma (100 μL) (Pigliasco et al., 2024). This provides important technical support for future studies on the plasma concentration-response relationship of fenfluramine and for individualized treatment.

## 5 Limitations and future directions

Although many reports were gathered from the FAERS database for this study in order to assess the AEs of fenfluramine from a variety of angles, there are still certain restrictions. Firstly, the AEs come from a range of sources and were freely reported in the FAERS database. Some important factors, such gender and indication, were absent from the data even after subgroup analyses were performed. The results may have been affected by this absence as well as problems with overreporting and underreporting, which might have influenced the disproportionality report’s measurement. Secondly, disproportionality studies cannot quantify risk or show causality, which can only evaluate signal strength and establish statistical relationships. Disproportionality analyses measures cannot estimate incidence or necessarily account for a causal association but only facilitate the identification of AE. Thirdly, over 60% of cases were submitted by consumers rather than physicians, which may affect the reliability of some signals. We conducted a sensitivity analysis to reduce the bias in this manuscript, but we still need to discuss these results with caution. Further clinical trial assessments are necessary to validate these relationships, and we need to interpret the results of these analyses more cautiously in light of the aforementioned limitations as well as other potential confounders and biases. In order to better evaluate the safety and possible dangers of fenfluramine, future research should think about using more stringent prospective study designs that integrate clinical trials and epidemiological research approaches.

## 6 Conclusion

Our analysis focused on AEs associated with fenfluramine, along with other pertinent and significant AEs. The objective was to offer valuable perspectives for the surveillance and improvement of clinical drug safety. As the first long-term pharmacovigilance study of fenfluramine based on the FAERS database, we have identified several common and rare AEs, providing a solid scientific foundation for the safety assessment of fenfluramine. In addition to the typical side effects such as seizure, somnolence, lethargy, status epilepticus, balance disorder, drooling and sedation, it is important to pay attention to emerging risks such as pericardial effusion, crying, pneumonia, oxygen saturation decreased, muscle twitching, insomnia, aggression, agitation, mood swings, urinary retention and aortic dilatation. It is notable that aortic valve incompetence and epilepsy are more likely to occur in males and females are more prone to encountering nervous system adverse reactions after fenfluramine treatment. Medical staff should pay more attention to cardiac AEs on LGS patients and nervous system AEs on DS patients throughout the entire duration of fenfluramine treatment. Doctors should consider the balance of risk-benefit ratio based on the patient’s specific condition. Echocardiographic monitoring in LGS and neurobehavioral surveillance in DS are recommended when initiating fenfluramine therapy.

## Data Availability

The original contributions presented in the study are included in the article/Supplementary Material, further inquiries can be directed to the corresponding author.

## Abbreviations

LGS, Lennox-Gastaut syndrome; DS, Dravet syndrome; AE, adverse event; FAERS, Food and Drug Administration (FDA) Adverse Event Reporting System; ROR, Reporting Odds Ratio; BCPNN, Bayesian Confidence Propagation Neural Network; PRR, Proportional Reporting Ratio; MGPS, Multi-Item Gamma Poisson Shrinker; GTCS, generalized tonic-clonic seizures; ASM, anti-seizure drug; ACE, ADHD Child Evaluation; VHD, valvular heart disease; PAH, pulmonary arterial hypertension; PT, preferred term; SOC, system organ class; PS, primary suspected drug; TTO, time to onset; CI, confidence interval; GABAergic, γ-aminobutyric acid-ergic; 5-HT, serotonin; TGF-β, transforming growth factor-beta.
