# Supplementary material for: Pharmacovigilance study on old drugs repurposed for rare diseases across different indications: the case of fenfluramine
Source: Front Pharmacol. 2025 Sep 24;16:1682788. doi: 10.3389/fphar.2025.1682788 (PMC12504265; doi:10.3389/fphar.2025.1682788)
Supplement: Supplementary file 1 [file Table1.docx]

**Supplementary Table 1**. Top 50 frequency of adverse events at the PT level for fenfluramine

| SOC | PT (Preferred Term) | Case number | ROR (95%Cl) | IC (IC025) | PRR (χ^2^) | EBGM (EBGM05) |
| --- | --- | --- | --- | --- | --- | --- |
| Cardiac Disorders | Tricuspid Valve Incompetence | 198 | 269.1(231.8-312.41) | 7.87(7.65) | 263.72(45979.11) | 234.08(206.61) |
|  | Mitral Valve Incompetence | 158 | 136.4(115.99-160.41) | 6.98(6.74) | 134.23(19626.28) | 126.13(110.13) |
|  | Aortic Valve Incompetence | 81 | 195.34(155.43-245.51) | 7.47(7.14) | 193.75(14205.52) | 177.28(146.42) |
|  | Pulmonary Valve Incompetence | 78 | 622.31(482.92-801.93) | 8.89(8.53) | 617.4(36989.58) | 475.99(384.99) |
|  | Mitral Valve Thickening | 32 | 842.76(558.64-1271.38) | 9.22(8.65) | 840.03(19085.99) | 598.14(424.02) |
|  | Pericardial Effusion | 19 | 5.79(3.69-9.09) | 2.53(1.88) | 5.78(75) | 5.77(3.96) |
| General Disorders and Administration Site Conditions | Crying | 33 | 9.04(6.42-12.73) | 3.17(2.67) | 9.01(234.12) | 8.98(6.74) |
| Infections And Infestations | Pneumonia | 119 | 2.51(2.09-3) | 1.31(1.05) | 2.49(106.3) | 2.49(2.14) |
|  | Viral Infection | 35 | 7.59(5.44-10.59) | 2.92(2.43) | 7.57(198.87) | 7.54(5.71) |
|  | Rhinovirus Infection | 34 | 41.54(29.57-58.37) | 5.34(4.85) | 41.4(1314.4) | 40.61(30.56) |
|  | Gastroenteritis Viral | 21 | 7.91(5.15-12.15) | 2.98(2.36) | 7.9(126.1) | 7.87(5.5) |
|  | Ear Infection | 21 | 4.5(2.93-6.9) | 2.16(1.55) | 4.49(56.84) | 4.48(3.13) |
|  | Pneumonia Aspiration | 20 | 5.66(3.65-8.78) | 2.49(1.86) | 5.65(76.31) | 5.64(3.9) |
|  | Pharyngitis Streptococcal | 18 | 10.46(6.58-16.62) | 3.38(2.71) | 10.44(152.88) | 10.39(7.05) |
|  | Respiratory Syncytial Virus Infection | 16 | 9.01(5.51-14.72) | 3.16(2.46) | 8.99(113.18) | 8.96(5.94) |
| Investigations | Weight Decreased | 200 | 4.67(4.06-5.38) | 2.2(1.99) | 4.6(564.72) | 4.59(4.08) |
|  | Echocardiogram Abnormal | 63 | 387.87(296.2-507.91) | 8.34(7.96) | 385.4(20369.33) | 325.16(259.48) |
|  | Oxygen Saturation Decreased | 32 | 2.97(2.1-4.2) | 1.56(1.06) | 2.96(41.51) | 2.96(2.21) |
| Metabolism And Nutrition Disorders | Decreased Appetite | 319 | 8.97(8.02-10.03) | 3.12(2.95) | 8.71(2175.56) | 8.68(7.9) |
| Musculoskeletal And Connective Tissue Disorders | Muscle Twitching | 20 | 7.1(4.57-11.01) | 2.82(2.19) | 7.08(104.18) | 7.06(4.89) |
| Nervous System Disorders | Seizure | 1061 | 55.53(52.07-59.22) | 5.6(5.51) | 49.67(49523.94) | 48.53(45.99) |
|  | Somnolence | 247 | 9.2(8.11-10.45) | 3.16(2.98) | 9(1753.59) | 8.96(8.06) |
|  | Generalized Tonic-Clonic Seizure | 114 | 41.75(34.65-50.31) | 5.34(5.07) | 41.28(4394.57) | 40.49(34.65) |
|  | Lethargy | 100 | 14.93(12.25-18.19) | 3.88(3.59) | 14.79(1277.2) | 14.69(12.45) |
|  | Tremor | 68 | 3.32(2.61-4.21) | 1.72(1.37) | 3.3(109.29) | 3.3(2.7) |
|  | Seizure Cluster | 67 | 518.12(396.22-677.54) | 8.69(8.3) | 514.61(27517.39) | 412.5(329.57) |
|  | Epilepsy | 63 | 13.6(10.6-17.43) | 3.75(3.38) | 13.52(725.82) | 13.44(10.91) |
|  | Atonic Seizures | 55 | 651.69(481.14-882.71) | 8.95(8.52) | 648.07(27073.65) | 494(383.24) |
|  | Status Epilepticus | 51 | 33.6(25.46-44.33) | 5.04(4.64) | 33.43(1579.13) | 32.91(26.1) |
|  | Hypersomnia | 48 | 11.52(8.67-15.31) | 3.51(3.1) | 11.47(456.33) | 11.41(8.99) |
|  | Balance Disorder | 37 | 3.16(2.29-4.37) | 1.66(1.19) | 3.15(54.37) | 3.15(2.4) |
|  | Petit Mal Epilepsy | 36 | 61.62(44.21-85.88) | 5.9(5.42) | 61.4(2077.46) | 59.66(45.19) |
|  | Drooling | 35 | 37.12(26.56-51.89) | 5.18(4.7) | 36.99(1204.39) | 36.36(27.48) |
|  | Myoclonic Epilepsy | 30 | 156.79(108.13-227.36) | 7.18(6.65) | 156.32(4305.3) | 145.43(106.57) |
|  | Sedation | 29 | 7.93(5.5-11.42) | 2.98(2.45) | 7.91(174.34) | 7.88(5.81) |
|  | Change In Seizure Presentation | 28 | 117.4(80.19-171.88) | 6.79(6.24) | 117.07(3050.1) | 110.87(80.59) |
|  | Partial Seizures | 28 | 38.16(26.24-55.48) | 5.22(4.68) | 38.05(992.06) | 37.38(27.33) |
|  | Tonic Convulsion | 23 | 136.99(89.79-208.99) | 7(6.39) | 136.67(2906.11) | 128.28(90.09) |
|  | Dyskinesia | 23 | 4.36(2.89-6.56) | 2.12(1.53) | 4.35(59.2) | 4.34(3.08) |
| Psychiatric Disorders | Insomnia | 110 | 3.24(2.68-3.91) | 1.68(1.41) | 3.21(168.12) | 3.21(2.74) |
|  | Aggression | 81 | 16.67(13.39-20.77) | 4.04(3.72) | 16.54(1174.25) | 16.42(13.67) |
|  | Abnormal Behavior | 59 | 19.16(14.82-24.78) | 4.24(3.86) | 19.06(1000.49) | 18.89(15.23) |
|  | Agitation | 58 | 7.56(5.83-9.78) | 2.91(2.53) | 7.52(326.75) | 7.49(6.04) |
|  | Irritability | 57 | 9.54(7.35-12.39) | 3.24(2.86) | 9.49(431.36) | 9.45(7.6) |
|  | Behavior Disorder | 49 | 30.88(23.27-40.97) | 4.92(4.51) | 30.73(1388.91) | 30.29(23.91) |
|  | Anger | 26 | 7.29(4.96-10.72) | 2.86(2.3) | 7.27(140.27) | 7.25(5.25) |
|  | Mood Swings | 25 | 8(5.4-11.85) | 2.99(2.42) | 7.98(152.12) | 7.95(5.72) |
| Renal and Urinary Disorders | Urinary Retention | 21 | 4.62(3.01-7.1) | 2.2(1.59) | 4.61(59.35) | 4.61(3.22) |
| Respiratory, Thoracic and Mediastinal Disorders | Pulmonary Arterial Hypertension | 19 | 11.08(7.06-17.4) | 3.46(2.81) | 11.06(173) | 11.01(7.55) |
| Vascular Disorders | Aortic Dilatation | 32 | 240.35(166.6-346.74) | 7.75(7.22) | 239.57(6815.3) | 214.87(158.12) |

**Supplementary Table 2**. Sensitivity analysis of new potential AEs through only choosing reports by medical staffs (including health professional, physician and pharmacist)

| PT (Preferred Term) | Case number | ROR (95%Cl) | IC (IC025) | PRR (χ^2^) | EBGM (EBGM05) |
| --- | --- | --- | --- | --- | --- |
| Pneumonia | 32 | 2.12(1.49-3) | 1.07(0.57) | 2.1(18.63) | 2.1(1.57) |
| Aortic dilatation | 22 | 500.7(320.35-782.59) | 8.77(8.14) | 496.68(9596.02) | 438.05(301.46) |
| Insomnia | 20 | 2.98(1.92-4.62) | 1.57(0.93) | 2.96(26.03) | 2.96(2.05) |
| Agitation | 12 | 4.92(2.79-8.68) | 2.29(1.49) | 4.9(37.27) | 4.9(3.05) |
| Aggression | 11 | 8.01(4.43-14.49) | 2.99(2.16) | 7.98(67.04) | 7.96(4.85) |
| Pericardial effusion | 11 | 8.77(4.85-15.86) | 3.12(2.29) | 8.74(75.23) | 8.72(5.31) |
| Oxygen saturation decreased | 8 | 2.16(1.08-4.33) | 1.11(0.15) | 2.16(4.97) | 2.16(1.21) |
| Crying | 6 | 9.68 ( 4.34 - 21.59 ) | 3.27(2.18) | 9.66(46.49) | 9.64(4.93) |
| Muscle twitching | 5 | 7.79 ( 3.24 - 18.75 ) | 2.96(1.78) | 7.78(29.48) | 7.76(3.72) |
| Mood swings | 3 | 5.48 ( 1.76 - 17.01 ) | 2.45(1.01) | 5.47(10.96) | 5.47(2.12) |

Note: The red-marked numbers indicate that they do not meet the positive signal criteria (EBGM05 > 2).
